# SigWin-detector: a Grid-enabled workflow for discovering enriched windows of genomic features related to DNA sequences

**DOI:** 10.1186/1756-0500-1-63

**Published:** 2008-08-08

**Authors:** Márcia A Inda, Marinus F van Batenburg, Marco Roos, Adam SZ Belloum, Dmitry Vasunin, Adianto Wibisono, Antoine HC van Kampen, Timo M Breit

**Affiliations:** 1 Integrative Bioinformatics Unit, Swammerdam Institute for Life Sciences, Faculty of Science, University of Amsterdam, PO Box 94062, 1090 GB Amsterdam, The Netherlands; 2 Bioinformatics Laboratory, Academic Medical Center, Meibergdreef 9, 1105 AZ Amsterdam, The Netherlands; 3 Institute of Informatics, Faculty of Science, University of Amsterdam, Kruislaan 403, 1098 SJ Amsterdam, The Netherlands

## Abstract

**Background:**

Chromosome location is often used as a scaffold to organize genomic information in both the living cell and molecular biological research. Thus, ever-increasing amounts of data about genomic features are stored in public databases and can be readily visualized by genome browsers. To perform *in silico *experimentation conveniently with this genomics data, biologists need tools to process and compare datasets routinely and explore the obtained results interactively. The complexity of such experimentation requires these tools to be based on an e-Science approach, hence generic, modular, and reusable. A virtual laboratory environment with workflows, workflow management systems, and Grid computation are therefore essential.

**Findings:**

Here we apply an e-Science approach to develop SigWin-detector, a workflow-based tool that can detect significantly enriched windows of (genomic) features in a (DNA) sequence in a fast and reproducible way. For proof-of-principle, we utilize a biological use case to detect regions of increased and decreased gene expression (RIDGEs and anti-RIDGEs) in human transcriptome maps. We improved the original method for RIDGE detection by replacing the costly step of estimation by random sampling with a faster analytical formula for computing the distribution of the null hypothesis being tested and by developing a new algorithm for computing moving medians. SigWin-detector was developed using the WS-VLAM workflow management system and consists of several reusable modules that are linked together in a basic workflow. The configuration of this basic workflow can be adapted to satisfy the requirements of the specific *in silico *experiment.

**Conclusion:**

As we show with the results from analyses in the biological use case on RIDGEs, SigWin-detector is an efficient and reusable Grid-based tool for discovering windows enriched for features of a particular type in any sequence of values. Thus, SigWin-detector provides the proof-of-principle for the modular e-Science based concept of integrative bioinformatics experimentation.

## Findings

Genomic information is encoded in DNA and as such retained in a fairly steady configuration. In contrast to RNA, proteins, and metabolites, DNA is organized by a limited number of large chromosomes with relatively stable DNA sequences. Therefore, position in the DNA sequence, i.e., chromosome location, provides a convenient and essential scaffold for both the living cell and molecular biological research. In cells, for example, chromosomal organization is important for gene-transcription processes. Expression-profiling studies showed that gene expression is not only controlled at the level of individual genes, but also via autonomous regulation of chromosomal domains [1-5]. This suggests the existence of higher-order transcriptional regulatory mechanisms related to DNA organization or structures. The use of chromosomal organization in the life sciences is exemplified by the popularity of genome browsers that use chromosome location to map many genomic features, such as genes and their products, regulatory elements, gene expression, and epigenetic markers. The search for connections between genomic features is important in unraveling cellular mechanisms.

The pace at which omics experiments continuously keep producing large amounts of data about genomic features for an increasing number of sequenced genomes, creates a need for new high-throughput methods for identification of correlations between DNA related features [6-12]. Therefore, biologists would benefit from tools that could quickly identify enriched regions of genomic features. This would allow extensive, yet convenient *in silico *experimentation based on routinely processing and comparing multiple datasets. However, this requires these tools to be implemented in such a way that they deal with the many steps involved in this kind of experimentation. These include: acquiring the data from local or remote data repositories, converting it to the desired format, using it with the actual application that searches for the desired enrichment (possibly using Grid computation), visualizing the results, and comparing and/or integrating multiple datasets. Therefore, such a tool should be developed applying an e-Science approach [13-17]: it should be generic with respect to which data it can analyze, easy to adapt, and its parts should be reusable.

In an e-Science approach, a computational environment that provides transparent access to distributed data, adequate computational resources, as well as the necessary interfacing tools, is called a *virtual laboratory *(*VL*). *Workflow management systems *(*WMSs*, [18-21]) are an example of interfacing tooling that takes care of scheduling, keeps track of task executions, and provides the management framework necessary to develop applications inside a VL. WMSs can be used to design scientific workflows that automate *in silico *experimentation by providing a pipeline for streaming large quantities of data through various algorithms, applications and services.

This paper describes an e-Science based data integration and analysis tool: SigWin-detector. This application can detect clusters with increased (or decreased) density of a genomic feature in a DNA-related sequence in a fast and reproducible way. In the context of the development of a VL, our tool was implemented as a workflow running under WS-VLAM[20,21], a Grid-enabled WMS. A biological use case shows its relevance for biological research. SigWin-detector is based on a method previously used by Versteeg and coworkers [4] to detect *regions of increased and decreased gene expression *(*RIDGEs and anti-RIDGES*) in human transcriptome maps (HTM). We improved the original method by i) deriving an analytical formula for computing the new hypothesis probability distribution, which replaces the costly step of estimation by random sampling and ii) developing a new algorithm for computing moving medians. While these improvements radically increase the intrinsic efficiency of the method, implementing SigWin-detector using a generic e-Science approach with access to Grid resources broadens its applicability and makes it amenable to a wide spectrum of experiments on genomic features or in fact on any sequence of values.

### Significant windows and the mmFDR procedure

**Figure 1:**
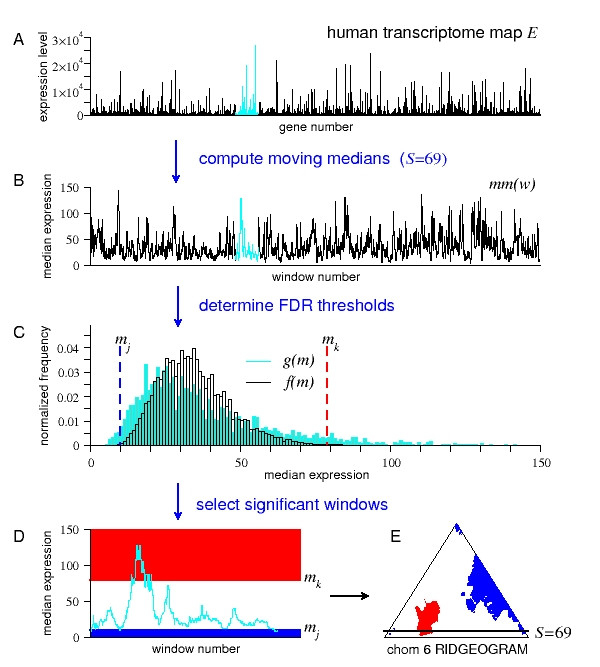
**Using a mmFDR method to detect RIDGEs in a human transcriptome map**. Schematic representation of the moving median false discovery rate (mmFDR) procedure identifying regions of high and low density of gene expression (RIDGEs and anti-RIDGEs, respectively) [4]. (A) Input sequence, a human transcriptome map (HTM), i.e., expression values of genes ordered by their chromosome location (cyan; chromosome 6). (B) *mm*(*w*), moving medians of the HTM for a given window size *S*. (C) Determination of the high and low mmFDR thresholds at a given level *α*: The high threshold *m*_*k *_is the smallest gene expression value for which the $\sum_{m\geq m_{k}}f(m)/\sum_{m\geq m_{k}}g(m)\leq \alpha$, here *f*(*m*) is the theoretical probability distribution of *mm*(*w*), and *g*(*m*) is the observed distribution of *mm*(*w*). (In [4], *f*(*m*) is estimated by simple sampling). Similarly, the low threshold *m*_*j *_is the largest gene expression value for which $\sum_{m\leq m_{j}}f(m)/\sum_{m\leq m_{j}}g(m)\leq \alpha$. (D) Selection of significant windows in chromosome 6: RIDGEs (in red) all windows for which the median gene expression is higher than or equal to *m*_*k*_; anti-RIDGEs (in blue) all windows for which the median gene expression is lower than or equal to *m*_*j*_. (E) Output RIDGEOGRAM of chromosome 6. Each row (y-axis) in the RIDGEOGRAM represents a window size, ranging from *S *= 3 to *S *= *M *(the number of genes on the chromosome). Each column (x-axis) represents a sliding window number, ranging from *w *= S/2 to *w *= *M*-*S*/2 (hence the triangular form). Color is used to mark window medians significantly above (red) or below (blue) the genome-wide median. The scheme shows median expression data for window size *S *= 69 and FDR thresholds level *α *= 5%.

Versteeg et al. [4] identified clusters where the median expression level of the genes involved is significantly higher than expected (RIDGEs), using a *moving median false discovery rate (mmFDR) *procedure (Figure 1). The mmFDR procedure identifies RIDGEs by testing the input gene-expression against the null hypothesis that the position of the genes on the chromosomes does not affect their expression levels. This same procedure can be used to identify *significant windows *(i.e., windows in the input sequence that have a median value that deviates significantly from expected, if assumed that the ordering of the numbers in the input sequence is random) related to any genomic feature mapped to DNA sequences. In an even wider scope, it can also be used to identify significant windows in any sequence of numbers.

### Avoiding permutations in the mmFDR procedure

Computationally, the most expensive step in the original mmFDR procedure is the repeated determination of medians over sliding windows of permutations of the input data to estimate the probability function corresponding to the null hypothesis. Our first improvement to the original method was to derive an exact formula for this distribution (see definitions and derivation in Additional file 1):

$$\hat{f}(r)=\frac{\left(\begin{array}{c} r-1\\ K \end{array}\right)\left(\begin{array}{c} N-r\\ S-K-1 \end{array}\right)}{\left(\begin{array}{c} N\\ S \end{array}\right)}=\frac{\left(\begin{array}{c} r-1\\ (S-1)/2 \end{array}\right)\left(\begin{array}{c} N-r\\ (S-1)/2 \end{array}\right)}{\left(\begin{array}{c} N\\ S \end{array}\right)}=\frac{S}{N}\frac{\left(\begin{array}{c} r-1\\ (S-1)/2 \end{array}\right)\left(\begin{array}{c} N-r\\ (S-1)/2 \end{array}\right)}{\left(\begin{array}{c} N-1\\ S-1 \end{array}\right)}.$$

This exact formula reduces the number of cycles of computing moving medians of an input sequence of approximately 25,000 entries from at least 5,000 to 1, giving SigWin-detector the efficiency it needs to be used routinely and for processing and comparing multiple datasets within minutes to hours, instead of days. This efficiency could not be if *f*(*m*) was estimated by sampling the permutation space **E**_*π*_, and counting the number of times *m *was the median value in any sliding window of size *S*.

### Speeding up the computation of moving medians

**Figure 2:**
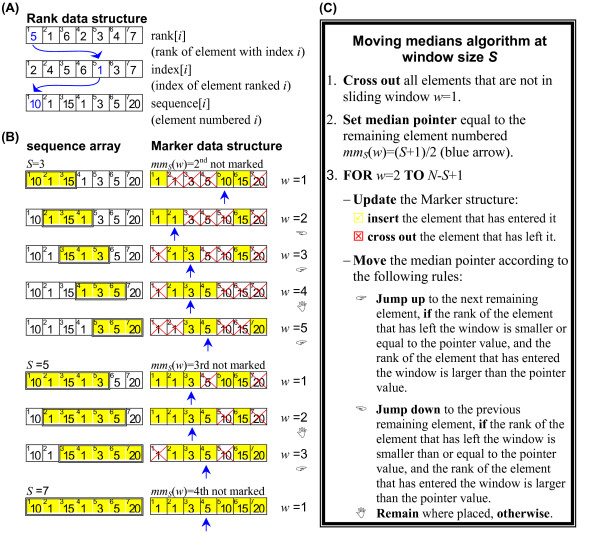
**Computing moving medians for many window sizes**. Description of our moving medians algorithm and data structures used. The figure illustrates a computation with input sequence size *N *= 7, and window sizes *S *= 3, 5, 7. (A) *Rank *data structure: used to store the input sequence. The Rank data structure gives access to the input sequence in its original and ranked order. It also allows fetching elements according to their rank. (B) *Marker *data structure: helps navigation through the sliding windows while keeping track of the median (or any other desired order-statistics). The Marker data structure is a Boolean array used to keep track of the elements that are inside a sliding window by means of crossing out the elements that are outside it. It also has a pointer that keeps track of the *i*th remaining element. This pointer is used to track the median. The Marker structure assumes the sequence is in ranked order. For example, if a sliding window of size 3 of a sequence of size 7 contains elements ranked 5, 1, and 6, the corresponding Marker structure has elements ranked 2, 3, 4, and 7 crossed out, and its median pointer points to element ranked 5. (C) Moving median algorithm for window size S. Our algorithm computes the moving medians for window sizes *S *= *Smin*, *Smin+*d*S,..., Smin+n*·d*S*, starting at *S *= *S*_*min*_. When the last sliding window of size *S *is reached, the algorithm proceeds to the next window size (*S*+d*S*) by inserting the elements that are in the first sliding window of size *S*+d*S *and crossing out the elements that were in the last sliding window of size *S *and setting the new position for the median pointer (which is element *mm*(*S+*d*S*) = (*S*+d*S*+1)/2). The algorithm stops after computing the medians for the largest window size.

Although we removed the need for computing moving medians over permutations of the input sequence, we still need to compute medians of windows sliding over the input sequence. We developed a new algorithm to compute those moving medians efficiently by exploiting the fact that moving medians for many window sizes must be computed simultaneously (Figure 2). This new algorithm is also suitable for computing any other order-statistics.

Additional Figure A1 (Additional file 2) shows a graph comparing our moving medians algorithm with the commonly used Hardle and Steiger's algorithm [22]. While the execution time of their algorithm increases with window size (for a fixed sequence size), the execution time of our algorithm decreases with window size (Figure A1, upper panel). Because SigWin-detector needs to compute moving medians for many window sizes, our algorithm has a clear advantage over Hardle and Steiger's algorithm. In Figure A1, the break-even point of the cumulative computation is for *S*_*max *_around 400. The efficiency of our method can be further improved by using a mixed algorithm that uses Hardle and Steiger's algorithm for small window sizes and our algorithm for large window sizes, or by employing a divide-and-conquer approach. For example, a two-phase algorithm would start by dividing the input sequence into chunks of size 2*M*, with *M *≥ 2*S*_*max*_, and applying the original algorithm to each chunk separately. Similarly, the second phase computes the medians for the missing sliding windows by dividing the sequence into chunks of the same size, but now using an offset *M*. This two-phase algorithm is also suitable for parallelization.

### Designing a Grid-enabled generic workflow

To broaden the applicability of the mmFDR procedure, we implemented SigWin-detector using an e-Science approach by implementing a general, reusable, and adaptable tool with access to Grid resources using the WS-VLAM workflow management system[20,21].

**Figure 3:**
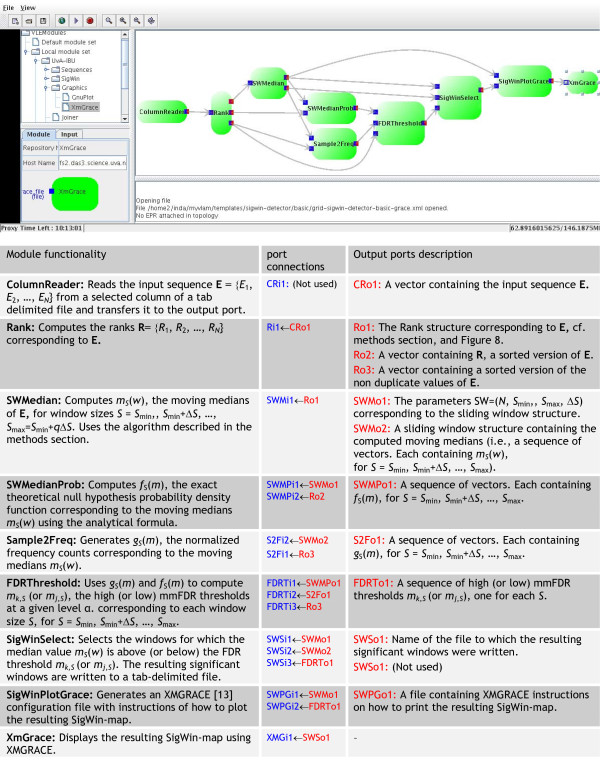
**SigWin-Detector basic workflow using the WS-VLAM workflow composer**. Upper: A snapshot of the workflow. Lower: Short description of the functionality of each module, port connections, and output ports. The ports are named by an abbreviation of the module name followed by 'i' or 'o' (input or output respectively) and the port number. Input ports are colored in blue and output ports in red. The ports are numbered in the same order they appear in the workflow.

First we split the procedure into a collection of workflow components (called modules), each module performing a specific task that may be fine-tuned using parameters. The modules exchange data with each other by means of input and output ports. We then can choose the appropriate modules and compose a workflow suited to our specific needs [16]. Figure 3 describes a basic workflow configuration of SigWin-detector.

The SigWin-detector Config-Basic1 workflow was tested on a Grid computer cluster composed of geographically distributed computational nodes: *Distributed ASCI Supercomputer 3 *(*DAS-3*, [23]). Additional Figure A2 (Additional file 2) presents wall clock execution times of the SigWin-detector Config-Basic1 workflow (Figure 3) for input sequences of various sizes.

The basic workflow can be altered by substituting, deleting, or adding modules. For example, we can extend the workflow to get the input sequence from a remote *uniform resource identifier *(*URI*)and then put the resulting SigWin-map back into it. We can modify the workflow to generate one SigWin-map per logical subsequence of the input sequence, instead of a single SigWin-map for the complete sequence [16]. We can also expand our workflow by computing significant windows for high median values (e.g., RIDGEs) and significant windows for low median values (e.g., anti-RIDGEs) simultaneously. The SigWin-detector workflow itself can be made into a "composite module" for more complex workflows. Furthermore, interconnection of WS-VLAM with the TAVERNA workbench [19] will permit the use of the existing TAVERNA components in connection with SigWin-detector. At the moment, Grid authentication prevents WS-VLAM workflows being used outside the Grid without the extra step of Grid certification. However, we are working on a Taverna workflow that encapsulates the SigWin detector, to be made available through the myExperiment webpage [24].

### Biological application: finding RIDGES in a human transcriptome map

**Figure 4:**
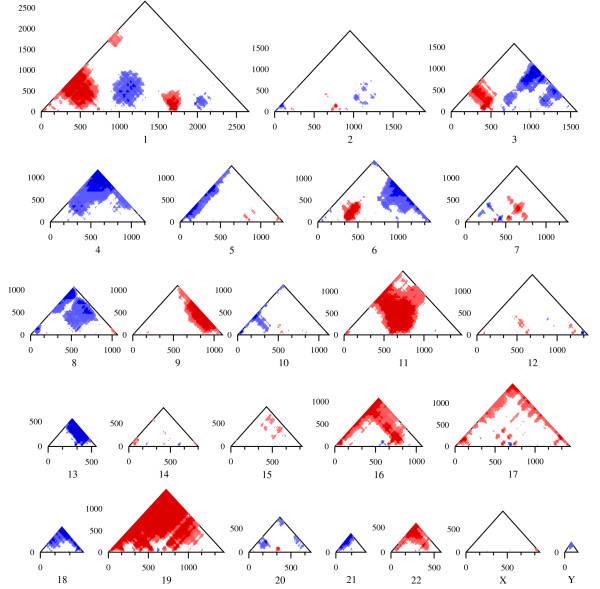
**Genome-wide RIDGES in a human transcriptome map (HTM)**. Genome-wide RIDGEOGRAMS per chromosome for the HTM based on the UCSC release hg18 [4]. The expression levels are mapped to gene number. Each RIDGEOGRAM displays a composite of both RIDGES (red-shades) and anti-RIDGEs (blue-shades) for different mmFDR rate levels: 10% (lighter shade), 5%, 1%, and 0.5% (darker shade). All the different window sizes are depicted because they give different specific results. In general, small windows suffer from noise and large windows suffer from lack of detail.

**Figure 5:**
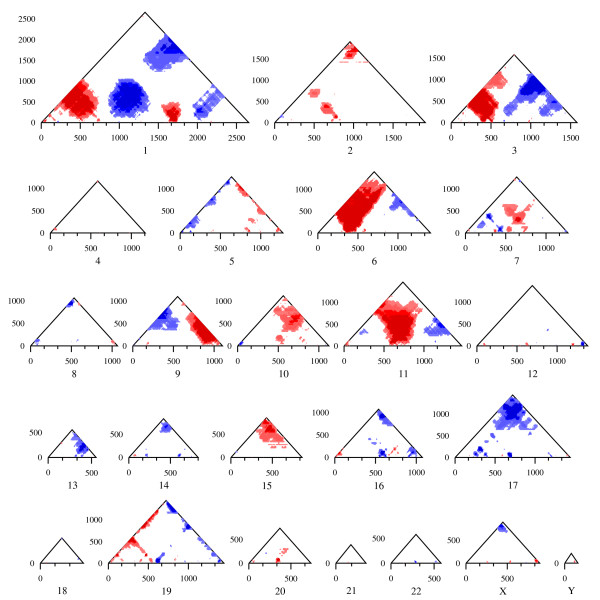
**Chromosome-specific RIDGES in a human transcriptome map (HTM)**. Chromosome-specific RIDGEOGRAMS per chromosome for the HTM based on the UCSC release hg18 [4]. The expression levels are mapped to gene number. Each RIDGEOGRAM displays a composite of both RIDGES (red-shades) and anti-RIDGEs (blue-shades) for different mmFDR rate levels: 10% (lighter shade), 5%, 1%, and 0.5% (darker shade).

Once we finished our basic SigWin-detector, we modified it (Additional file 3) for application in our biological use case that aims to find (anti-)RIDGES in transcriptome maps. Figures 4 and 5 show a series of RIDGEOGRAMS for gene expression data for a recent version of the human transcriptome map (HTM) based on the UCSC release hg18 [4], and Table 1 summarizes some RIDGE statistics. Each RIDGEOGRAM displays both RIDGEs (red-shades) and anti-RIDGEs (blue-shades), the different color shades representing different mmFDR threshold levels. The size of the resulting RIDGEOGRAMS is proportional to the number of genes on a chromosome. We determined i) genome-wide (anti-)RIDGEs, i.e., windows for which the median expression is significantly higher (lower) than expected by considering the whole genome gene expression profile in the mmFDR procedure (Figure 4), and ii) chromosome specific (anti-)RIDGEs, i.e., the same analysis, but considering only the specific chromosome gene expression profile (Figure 5). This distinction has a major effect on the outcome. If the expression values of the genes on a certain chromosome are typically significantly higher than the genome-wide values, then there are less chromosome specific than genome-wide RIDGEs (e.g., chromosome 19 in Figures 4 and 5 and Table 1). Conversely, if the expression values of the genes on a chromosome are typically significantly smaller than the genome-wide values, then there are more chromosome specific RIDGEs (e.g., chromosome 6 in Table 1 and Figures 4 and 5). In the case of anti-RIDGEs the opposite holds (e.g., chromosomes 17 in Table 1 and Figures 4 and 5). This example shows the importance of choosing the right sequence to compute the null hypothesis distribution. Based on the fact that chromosomes are separate molecules in a cell, one may favor the results from the individual chromosome SigWin-detector analysis to investigate potential higher-order gene expression regulatory mechanisms.

**Table 1:** HTM statistical data

				**RIDGEs**				**anti-RIDGEs**			
				**all window sizes**		**window sizes 19–59**		**all window sizes**		**window sizes 19–59**	

**chr**	**median**	**size**	**N**	**gw-R**	**chr-R**	**gw-R**	**chr-R**	**gw-aR**	**chr-aR**	**gw-aR**	**chr-aR**

Y	11	57772954	96	0	28	0	9	212	0	54	0
21	15	46944323	318	0	0	0	0	6957	0	266	0
18	16	76117153	488	0	8	0	0	23329	27	521	0
13	19	114142980	553	0	2	0	0	32667	10123	853	190
4	23	191273063	1172	0	323	0	0	121113	0	5	0
6	26	170899992	1406	28327	175351	873	1803	73404	12884	223	0
8	26	146274826	1067	213	61	32	0	83720	3110	176	20
10	26	135374737	1123	165	36813	9	0	9239	611	453	379
20	26.5	62435964	738	978	1350	292	538	4171	0	7	0
2	29	242951149	1908	1801	22546	247	52	2871	376	34	2
5	29	180857866	1276	722	8875	146	303	35406	10231	298	234
3	30	199501827	1581	47644	97097	806	1491	77920	85262	123	89
X	30	154913754	893	141	946	106	694	0	3667	0	0

*genome*	*33*	*3080419480*	*26740*	*1115947*	*767239*	*2511*	*9406*	*545680*	*554438*	*4832*	*6844*

1	34	247249719	2659	165349	154517	1611	1734	68461	271099	75	88
12	34	132349534	1382	1161	541	348	492	853	1262	530	661
7	35	158821424	1273	10549	19690	615	693	3614	5125	388	479
15	36	100338915	859	232	29110	0	0	0	0	0	0
11	38	134452384	1472	225772	150844	1508	814	0	14777	0	0
9	39	140273252	1103	62730	50105	214	571	7	27055	7	3
14	39	106368585	834	817	102	170	75	210	5968	186	489
17	44	78774742	1439	67267	0	1236	0	841	72325	434	1405
16	47	88827254	1075	107253	1388	1293	82	504	15676	63	1248
22	48	49691432	580	34220	0	255	0	12	242	12	219
19	52	63811651	1445	360606	17542	2748	55	169	14618	124	1338

chr: chromosome, median: median expression of all genes on a chromosome, size: chromosome size in base pairs, N: number of genes in chromosome, gw-R: number of genome-wide RIDGEs in a chromosome, chr-R: number of chromosome-specific RIDGEs in a chromosome, gw-aR: number of genome-wide anti-RIDGEs in a chromosome, chr-aR: number of chromosome-specific anti-RIDGEs in a chromosome.

The RIDGEOGRAMS shown in Figures 4 and 5 only take the ordering of the genes into account, and not their actual physical position in the chromosome. However, from a biological perspective it is likely that the higher order gene-expression mechanisms that underlie RIDGEs relate to an actual section of the chromosome rather than a cluster of genes just ordered by their chromosome location. So we used our SigWin-detector to take the physical gene position into account by subdividing the chromosomes in stretches of constant value (250 kb). If a stretch contains the beginning of one or more genes, their average expression value is assigned to that stretch of DNA. For this analysis we used the SigWin-detector Config-Sub2 with preprocessed HTM data and adapted parameters. The resulting RIDGEOGRAMS are proportional to the chromosome's size (Additional Figure A3, Additional file 2). The anti-RIDGEs show a lower cut-off caused by the many 0 values in the HTM. The results from the SigWin-detector analysis using chromosome position are substantially different to those using chromosome ordering. This application demonstrated that SigWin-detector is an e-Science tool that allows convenient in-silico experimentation. To prove that this tool is generic, we used our workflow to examine a simple sequential data set: an extended time series of hourly ground level ozone concentration measurements (Additional file 4).

## Availability and requirements

• **Project name**: SigWin-detector

• **Project home page: **

• **Programming language: **C++

• **Other requirements: **SigWin-detector needs the WS-VLAM workflow management system. WS-VLAM has a client distribution and site distribution.

*i. WS-VLAM client distribution: *The WS-VLAM composer, a graphical interface used for creating, modifying, and submitting workflows. Needs Java virtual machine (version1.5 or higher).

*ii. WS-VLAM site distribution: *The WS-VLAM engine, which is needed for running the workflows in a Grid. The WS-VLAM engine needs a GLOBUS GT4 (4.0.3) installation.

To download these WS-VLAM distributions (Additional file 5) go to , click the "Distributions" tab and follow the instructions in it.

## Competing interests

The authors declare that they have no competing interests.

## Authors' contributions

MAI carried out the entire research project and wrote the manuscript. MFvB participated in development of the statistical methods. MR was involved in the conceptualization of the analytical formula, in the e-Science approach, and in the coordination of the project. ASZB, DV, and WA worked on the development and support of WS-VLAM. AHvK developed the methods for the genomic mapping of expression data and was involved in the development of the statistical methods. TMB conceived the study, participated in its design and coordination and helped to draft the manuscript. All authors read and approved the final manuscript.

## Supplementary Material

Additional file 1 Derivation of the exact formula for the probability function *f*(*m*), and detailed description of the mmFDR-procedure. Click here for file

Additional file 2 Additional Figures. Click here for file

Additional file 3 Description of alternative SigWin-detector workflow configurations. Click here for file

Additional file 4 Applicability of SigWin-detector: periodic time series of air quality data. Click here for file

Additional file 5 This tar file contains the source files of the WS-VLAM modules needed to run the SigWin-detector workflow, and some examples. To uncompress use. ▪ tar -xvzf SigWin-VLAM.v1.1.tar.gz (Linux users). ▪ WinZip or a similar tool. Click here for file
