# Supplementary material for: SigWin-detector: a Grid-enabled workflow for discovering enriched windows of genomic features related to DNA sequences
Source: BMC Res Notes. 2008 Aug 8;1:63. doi: 10.1186/1756-0500-1-63 (PMC2533338; doi:10.1186/1756-0500-1-63)
Supplement: Additional file 1 — Derivation of the exact formula for the probability function f(m), and detailed description of the mmFDR-procedure. [file 1756-0500-1-63-S1.pdf]

Avoiding permutations in the moving median false discovery rate procedure

Supplementary information to manuscript: SigWin-detector: a Grid-enabled workflow for discovering enriched windows of genomic features related to DNA sequences

Authors: Márcia A. Inda, Marinus F. van Batenburg, Marco Roos, Adam S. Z. Belloum, Dmitry Vasunin, Adianto Wibisono, Antoine H. C. van Kampen, Timo M. Breit

Significant windows are detected by testing the input sequence against the following null hypothesis.

H_0: the position of the elements in a given sequence does not influence their values.

This null hypothesis can be tested with the following scenario: Let $\mathbf{E} = \{E_1, E_2, \cdots, E_N\}$ be a sequence of numbers of size N. Let $S \leq N$ represent a certain window size. Define the random variable M as the median expression value of a window of size S in the sample space ($\mathbf{\Omega}_S$, $\mathbf{E}_\pi$), the space of all possible (sliding) windows of size S that can be drawn from the space $\mathbf{E}_\pi = \{E_{\pi(1)}, E_{\pi(2)}, \cdots, E_{\pi(N)}\}$, the space of all possible permutations of the sequence $\mathbf{E}$. In this scenario, the probability function corresponding to the null hypothesis H_0 is given by

$$f(m) = P(M = m). \quad (1)$$

In this additional file we derive the exact formula for the probability function $f(m)$ for any given window size S. For sake of completeness, we also present a derivation of the *moving median false discovery rate (mmFDR)* procedure [1] in detail, which is depicted in Figure 1 of the main manuscript.

An exact formula for the probability function of moving medians

To derive $f(m)$, recall that the median value of a sequence of numbers of size S is equal to the element ranked $(S+1)/2$, if S is odd, or equal to the average of elements ranked $S/2$ and $S/2+1$, if S is even. Because computing median values depends on the ordering of the numbers in the sequence, we first convert the elements of the sequence into a list of ranks $\mathbf{R} = \{R_1, R_2, \cdots, R_N\}$ (here, $R_i = R(E_i) \in \{1, 2, \ldots, N\}$ is the rank of element E_i), then we compute the median value probabilities in the ranks domain, and finally we convert the ranks back to the actual expression values.

For simplicity, consider the case that all values are different and that S is odd. In this case, $f(m) = \hat{f}(R(m))$, where $\hat{f}(r)$ is the median value probability function in the domain of ranks. The problem of computing $\hat{f}(r)$ is equivalent to the order statistics problem of sampling without replacement from a population with finite support using equally likely probabilities [2, 3]. By definition, $\hat{f}(r)$ is the number of windows of size S that have median value equal to r (with $r \in \{1, 2, \ldots, N\}$) divided by $(N-S+1)\times N!$, the total number of windows of size S that can be drawn from the sample space $(\mathbf{\Omega}_S, \mathbf{E}_\pi)$.

As any window for which the median is equal to r must contain element r, $K=(S-1)/2$ elements smaller than r, and $S-K-1$ elements larger than r, it is easy to see that

$$\hat{f}(r) = 0, \qquad \text{if } r \le K \text{ or } r > N\text{-}K.$$

For $r \in \{K+1, K+2, \ldots, N\text{-}K\}$, there are:

$$\binom{r-1}{K}\binom{N-r}{S-K-1}\times S!\times(N-S)!\times(N-S+1) \tag{2}$$

windows for which the median is equal to r. *Proof:* there are $\binom{r-1}{K}\binom{N-r}{S-K-1}$ combinations of S elements containing K elements smaller than r and S-K-1 = K elements larger than r and smaller than or equal to N and S! ways of arranging those elements in a window of size S, giving

$$\binom{r-1}{K}\binom{N-r}{S-K-1}\times S!$$

combinations for which r is the median value. For each of those combinations there are $(N\text{-}S)!$ possible ways of arranging the remaining N-S elements of the sequence, multiplied by N-S+1 possible starting positions for each combination within the sequence.

Dividing (2) by the total number of windows gives the desired result:

$$\hat{f}(r) = \frac{\binom{r-1}{K}\binom{N-r}{S-K-1}}{\binom{N}{S}} = \frac{\binom{r-1}{(S-1)/2}\binom{N-r}{(S-1)/2}}{\binom{N}{S}} = \frac{S}{N}\,\frac{\binom{r-1}{(S-1)/2}\binom{N-r}{(S-1)/2}}{\binom{N-1}{S-1}}. \tag{3}$$

Note the similarity to the hyper-geometric distribution.

The case that S is even can be addressed with the same approach. In the case that not all values E_i are distinct, we simply rank the values that are the same consecutively

and sum the probabilities of all ranks that correspond to the same value. This formula is general and can be used for computing any other order statistic K, for $1 \le K \le S$.

The mmFDR procedure

Let $mm(w)$, with w=1, …, N–S+1, be the median expression in each (sliding) window w of size S of the input sequence **E**. As a first step to identify significant windows, each of the N–S+1 median expression values, $mm(w)$, have to be tested against the null hypothesis H_0. This multiple testing generates N–S+1 p-values (one for each sliding window):

$$p(mm(w)) = P(M \ge mm(w)) = \sum_{t \ge mm(w)} f(t), \qquad (4)$$

each p-value giving the probability that the random variable M takes values larger or equal to $mm(w)$, independently of the ordering of the sequence. To account for multiple testing, the mmFDR procedure uses these p-values as input to the procedure described by Benjamini and Hochberg [4], which controls the FDR.

A reformulation of the FDR procedure

The *Benjamini and Hochberg (BH)* FDR procedure takes as input the N–S+1 p-values computed according to Eq. (4) and computes the desired cutoff p-value as follows:

1. Sort all p-values so that p_1 is the smallest and p_{N-S+1} is the largest, and define $p_0 = 0$.
2. Find the largest k for which
   $$p_k \le \alpha \cdot k/(N-S+1). \qquad (5)$$
3. Define the cutoff p-value to be p_k.

Choosing p_k as the cutoff value means that the k windows for which $p(mm(w)) \le p_k$ will be considered significant. Defining m_k as the kth median value in order of magnitude (i.e., m_k is the median value corresponding to the kth p-value p_k). The median value m_k is called the FDR threshold, because all k windows with median value $mm(w) \ge m_k$ will be marked as significant or RIDGEs, while the windows with median value $mm(w) < m_k$ will not.

The mmFDR method is equivalent to the HB FDR controlling procedure applied to p-values for identifying significant moving medians, but it computes the FDR threshold m_k directly by comparing ratios of the tails of the probability

distribution function *f* divided by tails of the observed normalized histogram of *mm*(*w*):

$$g(m) = \frac{\text{number of times } m = mm(w), \text{ for } w = 1,2,\ldots, N-S+1}{N-S+1}. \quad (6)$$

To see how this approach works, note that k is equal to the number of times a median value $mm(w) \geq m_k$ in any of the N–S+1 windows of size S. Therefore, dividing k by N–S+1 gives

$$\frac{k}{N-S+1} = \sum_{m \geq m_k} g(m). \quad (7)$$

Substituting Eq. (4) and Eq. (7) into inequality (5) gives

$$\frac{\sum_{m \geq m_k} f(m)}{\sum_{m \geq m_k} g(m)} \leq \alpha, \quad (8)$$

where m_k is the smallest median expression value for which the above inequality holds.

Significant windows for low median values

It is also interesting to look for windows where the median values are lower than expected by the null hypothesis (regions of decreased density of gene expression – anti-RIDGEs, for the case of transcriptome maps). In this case, the low (anti-RIGDE) FDR threshold m_j will be the largest median value that satisfies

$$\frac{\sum_{m \leq m_j} f(m)}{\sum_{m \leq m_j} g(m)} \leq \alpha \quad (9)$$

References

1. Versteeg R, van Schaik BD, van Batenburg MF, Roos M, Monajemi R, Caron H, Bussemaker HJ, van Kampen AH: **The human transcriptome map reveals extremes in gene density, intron length, GC content, and repeat pattern for domains of highly and weakly expressed genes**. *Genome Res* 2003, **13**(9):1998-2004.
2. Arnolds BC, Balakrishnan N, Nagaraja H: *A first course in order statistics.* New York John Wiley 1992.
3. Evans DL, Leemis LM, Drew JH: **The distribution of order statistics for discrete random variables with applications to bootstrapping.** *Informs J Comput* 2006, **18:**19-30.
4. Benjamini Y, Hochberg Y: **Controlling the False Discovery Rate - a Practical and Powerful Approach to Multiple Testing**. *J Roy Stat Soc B Met* 1995, **57**(1):289-300.
