# Supplementary material for: SigWin-detector: a Grid-enabled workflow for discovering enriched windows of genomic features related to DNA sequences
Source: BMC Res Notes. 2008 Aug 8;1:63. doi: 10.1186/1756-0500-1-63 (PMC2533338; doi:10.1186/1756-0500-1-63)
Supplement: Additional file 2 — Additional Figures. [file 1756-0500-1-63-S2.pdf]

Additional Figures

Manuscript: SigWin-detector: a Grid-enabled workflow for discovering enriched windows of genomic features related to DNA sequences

Authors: Márcia A. Inda, Marinus F. van Batenburg, Marco Roos, Adam S. Z. Belloum, Dmitry Vasunin, Adianto Wibisono, Antoine H. C. van Kampen, Timo M. Breit

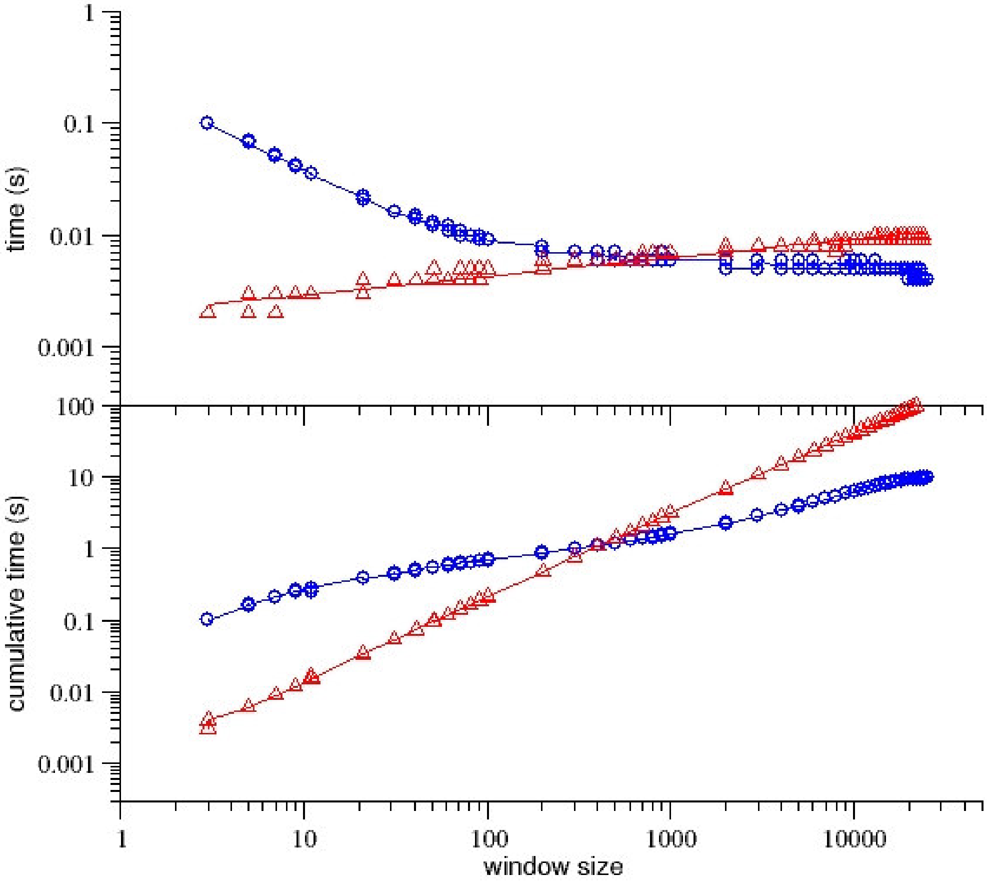

Figure A1 - Moving medians comparison

Upper: Time in seconds (s) to compute a moving median profile as a function of the window size for a sequence of size 25.000. Lower: Corresponding cumulative time to compute moving median profiles for a range of windows sizes S=3, 5, ..., S_{max} as a function of maximum window size S_{max}. Triangles: Hardle and Steiger's algorithm [26]. Circles: our algorithm.

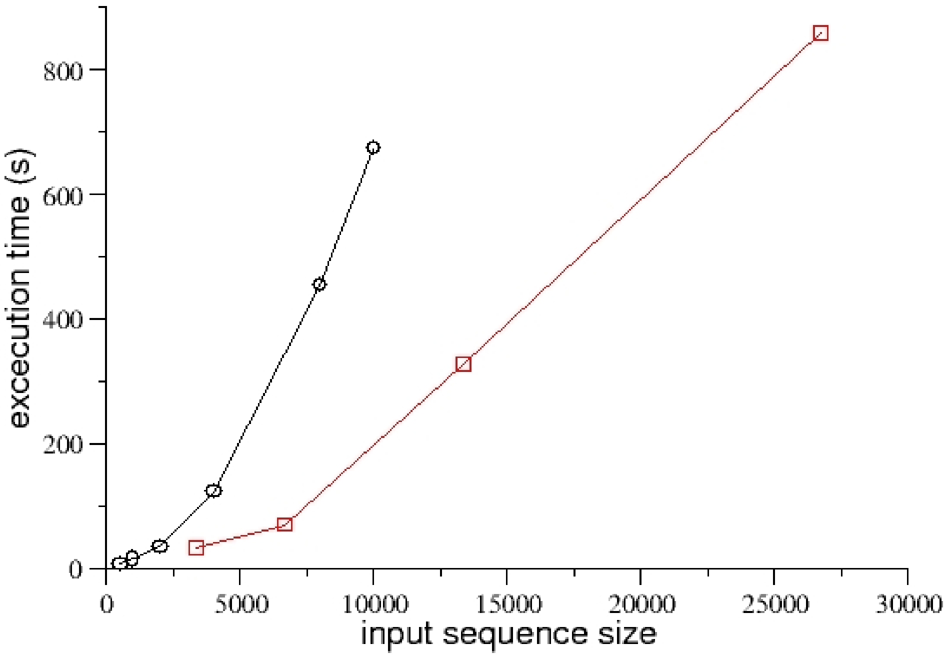

Figure A2 - SigWin-detector performance

Timing results of the SigWin-detector basic workflow as a function of the input sequence size *N* for different window ranges *S*=3, 5, ..., S_{max}. Squares: S_{max} ~ *N*. Circles line: S_{max} ~ *N*/10.

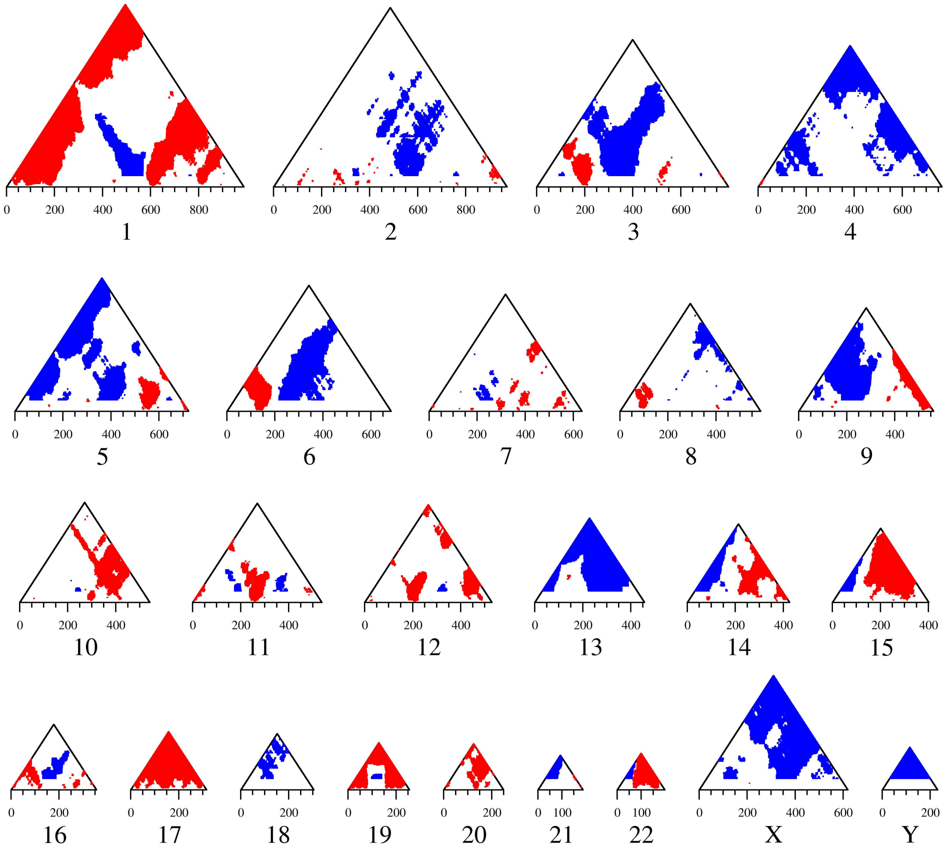

Figure A3 - Positional RIDGES in a human transcriptome map (HTM)

Positional genome-wide RIDGEOGRAMS per chromosome for the HTM based on the UCSC release hg18 [4] at 5% mmFDR level, made using SigWin-detector Config-Sub2 (additional information). The expression levels are mapped to chromosome position. RIDGES are in red, anti-RIDGES are in blue.
