# Supplementary material for: SigWin-detector: a Grid-enabled workflow for discovering enriched windows of genomic features related to DNA sequences
Source: BMC Res Notes. 2008 Aug 8;1:63. doi: 10.1186/1756-0500-1-63 (PMC2533338; doi:10.1186/1756-0500-1-63)
Supplement: Additional file 3 — Description of alternative SigWin-detector workflow configurations. [file 1756-0500-1-63-S3.pdf]

Alternative configurations for SigWin-detector workflow

Manuscript: SigWin-detector: a Grid-enabled workflow for discovering enriched windows of genomic features related to DNA sequences

Authors: Márcia A. Inda, Marinus F. van Batenburg, Marco Roos, Adam S. Z. Belloum, Dmitry Vasunin, Adianto Wibisono, Antoine H. C. van Kampen, and Timo M.Breit

Here we describe four alternative configurations for the SigWin-detector workflow. Configurations Basic1 and Basic2 are used in the case we want to compute one single SiqWin-map for the entire input data sequence. Configurations Sub1 and Sub2 are used in the case that the input data sequence can be subdivided into logical subsequences and we want to present the output in terms of those subsequences (see Figures 5 and 6 in the main paper), thus one SigWin-map per subsequence. For instance, a transcriptome map has chromosomes as logical subsequences; a time series may have years, months, days, etc, as logical subsequences. Configurations ending in 1 only compute one significant window type: windows with significantly high median values or windows with significantly low median value. Configurations ending in 2 compute both significant window types and plot them with different colors.

Contents:

SigWin-detector Config-Basic: Detects significant windows in a sequence. 2
SigWin-detector Config-Basic2: Detects significantly high median windows and significantly low median in a sequence. 3
SigWin-detector Config-Sub: Detects significant windows in a subsequence. 4
SigWin-detector Config-Sub2: Detects significantly high median windows and significantly low median windows for subsequences. 5
Module descriptions 6
Obtaining the software 7
See section Input file example 8
Output file example 8

SigWin-detector Config-Basic: Detects significant windows in a sequence.

Input: A space-delimited file with (at least) one column containing the input sequence **E**= {E_1, E_2, …, E_N}. A two-line header should precede the data. See section *Input file example*.

Output:

1. A file containing the detected significant windows for each label. Each data row represents a stretch of consecutive significant windows. Column 1 gives the window size and columns 2 and 3 give the first and last significant windows in the stretch. See section *Output file example*.
2. An XMGRACE configurations file with information on how to plot the resulting SigWin-map (Use: xmgrace -bat file.agrcmd)

Graphical output: An XMGRACE plot displaying the resulting SigWin-map.

Workflow:

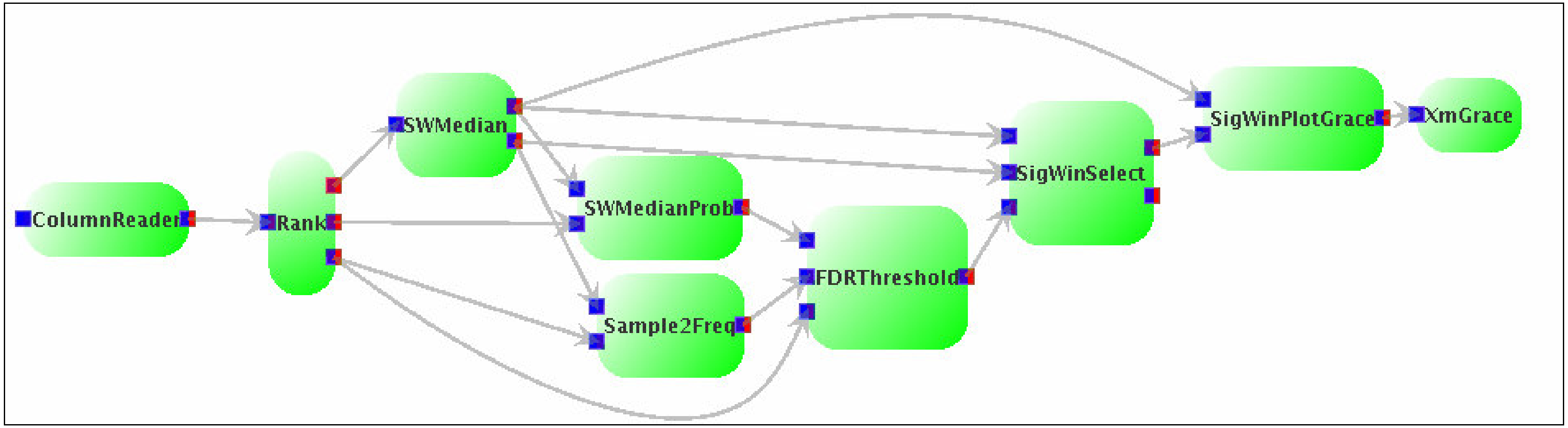

Parameters to set: (see section *module descriptions*)

***ColumnReader**:* file_name, column.

***SWMedian**:* min_window_size, max_window_size, step_size.

FDRThresold: FDR_level, threshold.

SigWinSelect: Output_file, threshold.

SigWin-detector Config-Basic2: Detects significantly high median windows and significantly low median in a sequence.

Input: A space-delimited file with (at least) one column containing the input sequence **E**= $\{E_1, E_2, \ldots, E_N\}$ See section *Input file example*.

Output:

1 A file containing the detected significantly high median windows for each label. See section *Output file example*.
2 A file containing the detected significantly low median windows for each label. See section *Output file example*.
3 An XMGRACE configurations file with information on how to plot the SigWin-maps. (Use: xmgrace -bat file.agrcmd)

Graphical output: An XMGRACE plot displaying the corresponding SigWin-maps.

Workflow:

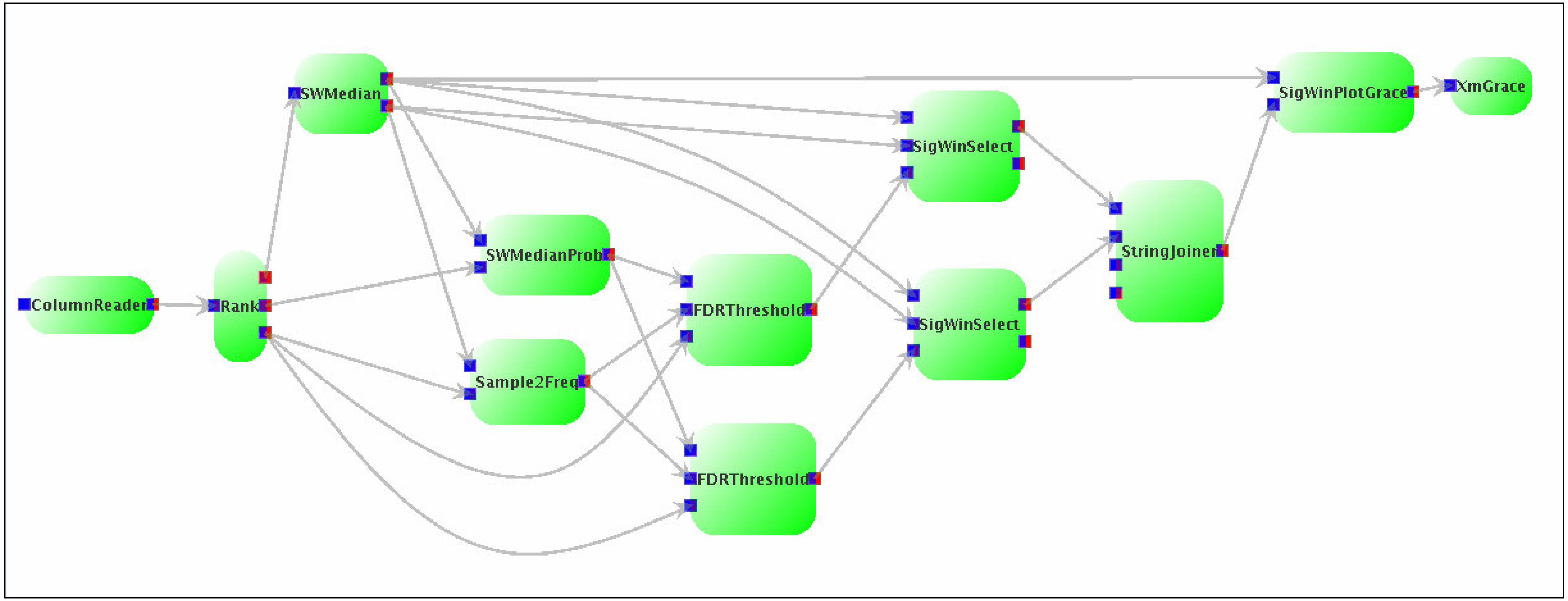

Parameters to set: (see section *module descriptions*)

***ColumnReader**:* file_name, column.

***SWMedian**:* min_window_size, max_window_size, step_size.

FDRThresold (upper): FDR_level, threshold=high. ***FDRThresold (lower):*** FDR_level, threshold=low.

SigWinSelect (upper): Output_file, threshold=high. ***SigWinSelect (lower):*** Output_file, threshold=low.

StringJoiner: n_streams=2

SigWin-detector Config-Sub: Detects significant windows in a subsequence.

Input: A space-delimited file with (at least) two columns. Column A: a sequence of labels **L**= {L_1, L_2, …, L_N}. Column B: the input sequence **E**= {E_1, E_2, …, E_N}. See section *Input file example*.

Output:

1 A file containing the detected significant windows for each label. See section *Output file example*.

2 An XMGRACE configuration file with information on how to plot the resulting SigWin-maps (Use: xmgrace -bat file.agrcmd)

Graphical output: An XMGRACE plot displaying the resulting SigWin-maps.

Workflow:

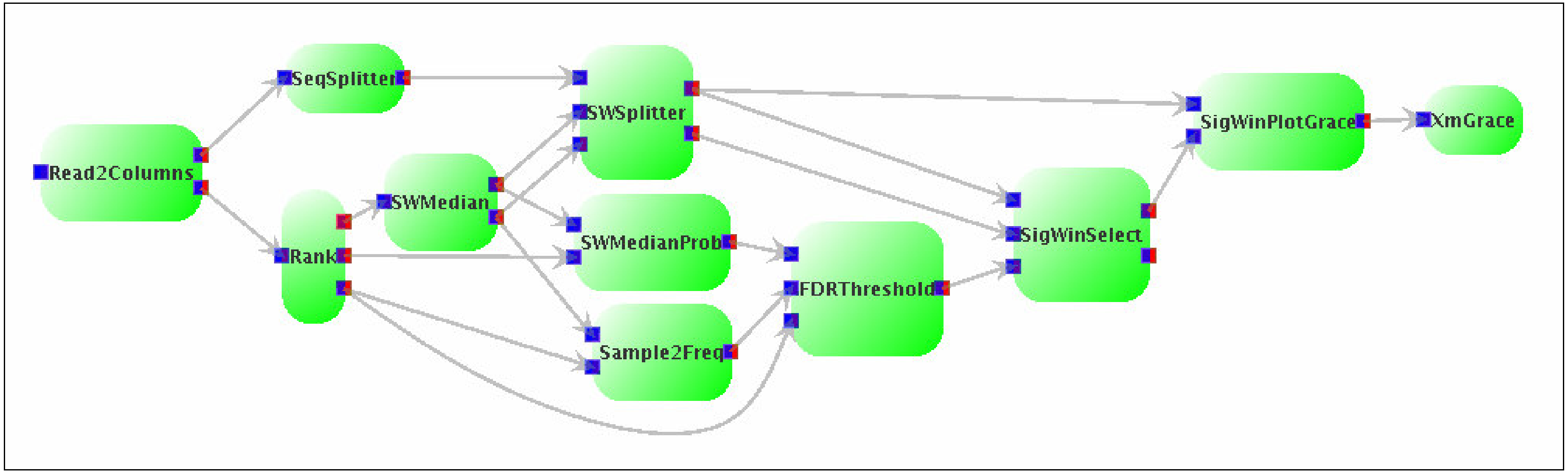

Parameters to set: (see section *module descriptions*)

***Read2Clumns**:* file_name, column1, column2.

***SWMedian**:* min_window_size, max_window_size, step_size.

FDRThresold: FDR_level, threshold.

SigWinSelect: Output_file, threshold.

SigWin-detector Config-Sub2: Detects significantly high median windows and significantly low median windows for subsequences.

Input: A space-delimited file with (at least) two columns. Column A: a sequence of labels L= {L_1, L_2, …, L_N}. Column B: the input sequence E= {E_1, E_2, …, E_N}. See section *Input file example*.

Output:

1 A file containing the detected significantly high median windows for each label. See section *Output file example*.
2 A file containing the detected significantly low median windows for each label. See section *Output file example*.
3 An XMGRACE configurations file with information on how to plot the SigWin-maps. (Use: xmgrace -bat file.agrcmd)

Graphical output: An XMGRACE plot displaying the corresponding SigWin-maps.

Workflow:

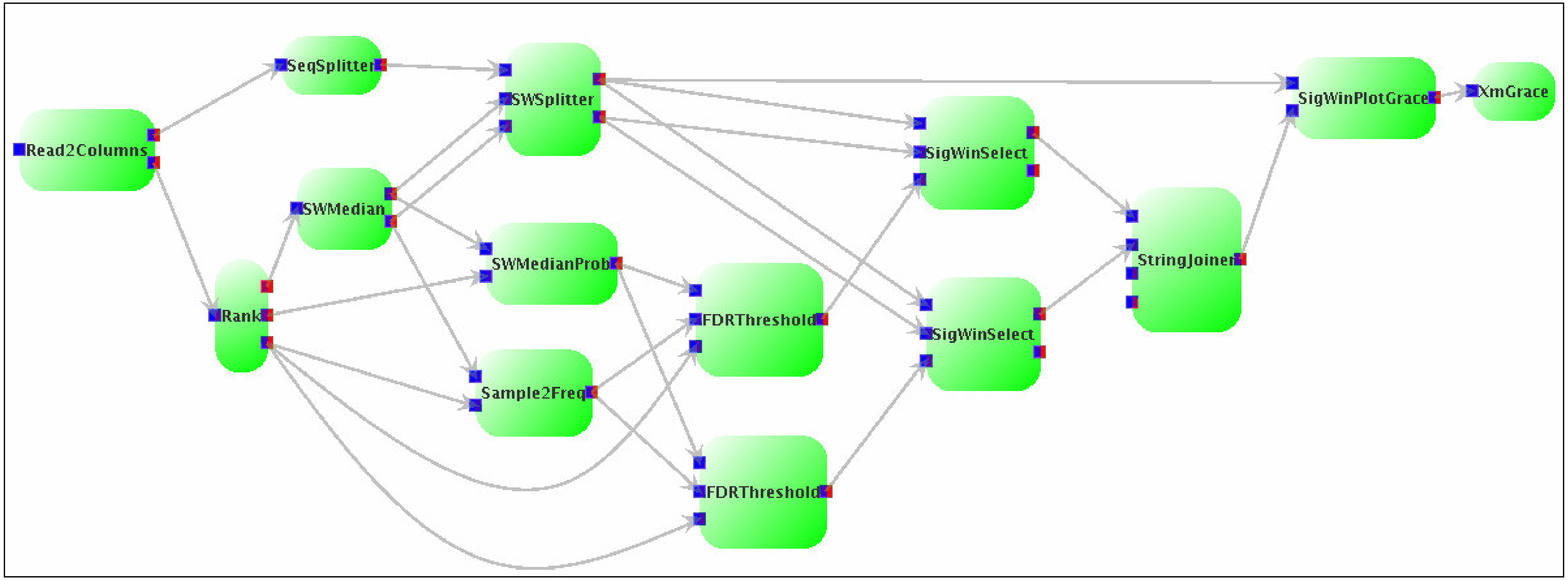

Parameters to set: (see section *module descriptions*)

***Read2Clumns*:** file_name, column1, column2.

***SWMedian*:** min_window_size, max_window_size, step_size.

FDRThresold (upper): FDR_level, threshold=high. ***FDRThresold (upper):*** FDR_level, threshold=low.

SigWinSelect (lower): Output_file, threshold=high. ***SigWinSelect (lower):*** Output_file, threshold=low.

StringJoiner: n_streams=2

Module descriptions

A short description of each module containing the modules functionality, most used parameters, input ports and output ports is given below. Only the parameters that must be set before execution are listed (the other parameters may keep their default value). The port connections are given in the workflow diagrams. The contents of the input ports are the same as the contents of the corresponding output ports. The ports are named by an abbreviation of the module name followed by 'i' or 'o' (input or output respectively) and the port number. Input ports are colored in blue and output ports in red. The ports are numbered in the same order as they appear in the workflow diagrams.

Module functionality	Parameters	Input ports	Output ports description
**ColumnReader**: Reads the input sequence **E**= $\{E_1, E_2, \ldots, E_N\}$from a tab delimited file and transfers it to the output port.	*file_name*: Input file name<br>*column*: Column number	CRi1 (Not used)	CRo1: A vector containing the input sequence **E**
**Read2Columns**: Reads two columns from a tab delimited file and transfers it to the output ports. Column A: a sequence of labels **L**= $\{L_1, L_2, \ldots, L_N\}$. Column B: the input sequence **E**= $\{E_1, E_2, \ldots, E_N\}$.	*file_name*: Input file name<br>*column1*: Column A number<br>*column2*: Column B number	R2Ci1 (Not used)	R2Co2: A vector containing the labels **L.**<br>R2Co1: A vector containing the input sequence **E**
**SeqSplitter:** Computes labeled subintervals from **L.** A labeled subinterval is determined by consecutive labels with the same value. E.g., **L**={a, a, b, b, b} produces two subintervals I_1= (a, 1, 2) and I_2=(b, 3, 5), defined by the triple (label, start, end).	-	SSPi1	SSPo1: A vector containing the labeled subintervals **I**=$\{I_1, I_2, \ldots\}$.
**Rank:** Computes the ranks **R**= $\{R_1, R_2, \ldots, R_N\}$ corresponding to **E.**	-	Ri1	Ro1: The Rank structure corresponding to **E**.<br>Ro2: A vector containing **R**, a sorted version of **E**.<br>Ro3: A vector containing a sorted version of the non duplicate values of **E**.
**SWMedian:** Computes $m_S(w)$, the moving medians of **E,** for window sizes $S = S_{min}, S_{min}+\Delta S, \ldots, S_{max} = S_{min}+q\Delta S$.	*min_window_size:* S_{min} (odd)<br>*max_window_size:* Approximate value for S_{max}<br>*step_size:* ΔS (even)	SWMi1	SWMo1: The parameters SW=$(N, S_{min}, S_{max}, \Delta S)$ corresponding to the sliding window structure.<br>SWMo2: A sliding window structure containing the computed moving medians (i.e., a sequence of vectors. Each containing $m_S(w)$, for $S = S_{min}, S_{min}+\Delta S, \ldots, S_{maz}$).
**SWSplitter:** Splits the input sliding window structure. Each new substructure corresponds to a labeled subinterval. E.g., sliding window SW=(N=30, S_{min},=1, S_{max} =15, ΔS=2) and intervals I_1= (a, 1, 12) and I_2=(b, 13, 30) produce sliding window substructures SW_1=(12, 1, 11, 2) and SW_1=(18, 1, 15, 2).	-	SWSPi1<br>SWSPi2<br>SWSPi3	SWSPo1: A vector containing the parameters for each sliding window substructure **SW**=$\{SW_1, SW_2, \ldots\}$.<br>SWSPo2: The corresponding sliding window substructures

Module functionality	Parameters	Input ports	Output ports description
**SWMedianProb:** Computes $f_S(m)$, the exact theoretical null hypothesis probability density function corresponding to the moving medians $m_S(w)$.		SWMPi1<br>SWMPi2	SWMPo1: A sequence of vectors. Each containing $f_S(m)$, for $S = S_{min}, S_{min}+\Delta S, \ldots, S_{min}+q\Delta S$.
**Sample2Freq:** Generates $g_S(m)$, the normalized frequency counts corresponding to the moving medians $m_S(w)$.		S2Fi2<br>S2Fi1	S2Fo1: A sequence of vectors. Each containing $g_S(m)$, for $S = S_{min}, S_{min}+\Delta S, \ldots, S_{min}+q\Delta S$.
**FDRThreshold:** Uses $g_S(m)$ and $f_S(m)$ to compute $m_{k,S}$ (or $m_{j,S}$), the high (or low) mmFDR thresholds at a given level α. corresponding to each window size S, for $S = S_{min}$, $S_{min}+\Delta S, \ldots, S_{min}+q\Delta S$.	*FDR_level*: α (0 to 1)<br>*Threshold*: high or low	FDRTi1<br>FDRTi2<br>FDRTi3	FDRTo1: A sequence of high (or low) mmFDR thresholds $m_{k,S}$ (or $m_{j,S}$), one for each S.
**SigWinSelect:** Selects the windows for which the median value $m_S(w)$ is above (or below) the FDR threshold $m_{k,S}$ (or $m_{j,S}$). The resulting significant windows are written to a tab-delimited file.	*Output_file*: output file name.<br>*Threshold*: high or low	SWSi1<br>SWSi2<br>SWSi3	SWSo1: Name of the file to which the resulting significant windows were written.<br>SWSo1: (Not used)
**StringJoiner:** Concatenate up to four strings and send then to the output port. In the SigWin-detector config-HT workflow: the strings are the names of the files containing the detected significantly high median windows and the significantly low median windows respectively.	*n_streams*: number of input ports to use (1 to 4).	SJi1<br>SJi2<br>SJi3 (Not used)<br>SJi4(Not used)	SJo1: The concatenated string.
**SigWinPlotGrace** Generates an XMGRACE [13] configuration file with instructions of how to plot the resulting SigWin-map.		SWPGi1<br>SWPGi2	SWPGo1: A file containing XMGRACE instructions on how to print the resulting SigWin-map.
**XmGrace** Displays the resulting SigWin-map using XMGRACE.		XMGi1	-

Obtaining the software

The source code for SigWin-detector modules can be downloaded from http://mad-db.science.uva.nl/projects/sigwin/. SigWin-detector runs under the WS-VLAM workflow management system. The client application of WS-VLAM can be downloaded and installed on any desktop machine, requiring only a Java virtual machine (version1.5 or higher). The current version of the client application, which is available from the WS-VLAM web site (http://staff.science.uva.nl/~gvlam/wsvlam/), still has some limitations. In the current version, workflow execution can only be done from a Linux machine. Because WS-VLAM was originally designed to work in a Grid environment, it uses Grid credentials for user authentication and authorization. Only users with valid Grid credentials can use WS-VLAM to run workflows on Grid computing resources. We are currently working on a method that will allow new users to bypass the process of requesting a new certificate, which takes a few days. A user with Grid credentials creates a valid Proxy using WS-VLAM and sends a link to the new user who can, in turn, use that link to execute workflows on Grid computing resources.

See section Input file example:

```
#size=26740 ncols=2 descr=hg18-htm
#chrom expression
1	10.0
1	13.0
1	286.0
1	0
…
1	46.0
2	37.0
2	5.0
2	77.0
…
24	7.0
24	74.0
24	96.0
24	1.0
```

Output file example:

```
#Windows beneath FDRThreshold for hg18-htm:1
#printing a point at wMin to get correct number of sets
1 1 1
#windowSize first last
25 210 221
25 994 996
27 212 220
…

#Windows beneath FDRThreshold for hg18-htm:2
#printing a point at wMin to get correct number of sets
1 1 1
#windowSize first last
29 18 21
47 30 31
49 29 32
51 29 32
…
```
