# Supplementary material for: SigWin-detector: a Grid-enabled workflow for discovering enriched windows of genomic features related to DNA sequences
Source: BMC Res Notes. 2008 Aug 8;1:63. doi: 10.1186/1756-0500-1-63 (PMC2533338; doi:10.1186/1756-0500-1-63)
Supplement: Additional file 4 — Applicability of SigWin-detector: periodic time series of air quality data. [file 1756-0500-1-63-S4.pdf]

Applicability of SigWin-detector: periodic time series of air quality data

Supplementary information to manuscript: SigWin-detector: a Grid-enabled workflow for discovering enriched windows of genomic features related to DNA sequences

Authors: Márcia A. Inda, Marinus F. van Batenburg, Marco Roos, Adam S. Z. Belloum, Dmitry Vasunin, Adianto Wibisono, Antoine H. C. van Kampen, Timo M. Breit

To prove that SigWin-detector is a generic tool, we used our workflow to examine a simple sequential data set: a time series of hourly ground level ozone concentration measurements in Amsterdam, the Netherlands, for the years 1997 up to 2004 [1]. Figures A4 and A5 present results obtained with SigWin-detector Config-Basic1 (Additional File 3), with FDR level at 5% for data from a period of several years, individual years, and individual months. The results of the eight-year period showed a checkerboard pattern that characterizes periodic data [2]. Thus, a large number of significant windows correspond to the warmer months of the year for window sizes smaller than one year. This was expected, given the periodic nature of the data series and the relationship between ozone production and sunlight. The larger number of significant windows at the end of the period indicates an overall increase in the ozone concentration over the years.

A close comparison of the results from the analysis of a period of years and the individual years showed little difference (Figure A4). This indicates that the null hypothesis distributions used to compute the mmFDR thresholds is similar in both cases. However, the results from the SigWin-detector analysis of one year are completely different to those of the individual months of that same year (Figure A5). The periodicity of the data set ensures that the null hypothesis distribution of medians remains relatively the same whether considering one or a multiple number of complete periods (i.e. years, Figure A4). But it changes drastically when considering any incomplete period (e.g. a month, Figure A5). In general, if the values in a subsequence are significantly higher than the values in the complete sequence, the mmFDR thresholds tend to be higher, thus resulting in less significant windows (April to September, Figure A5). Conversely, if the values in a subsequence are significantly lower, the mmFDR thresholds tend to be lower and more significant windows will be detected (October to February, Figure A4).

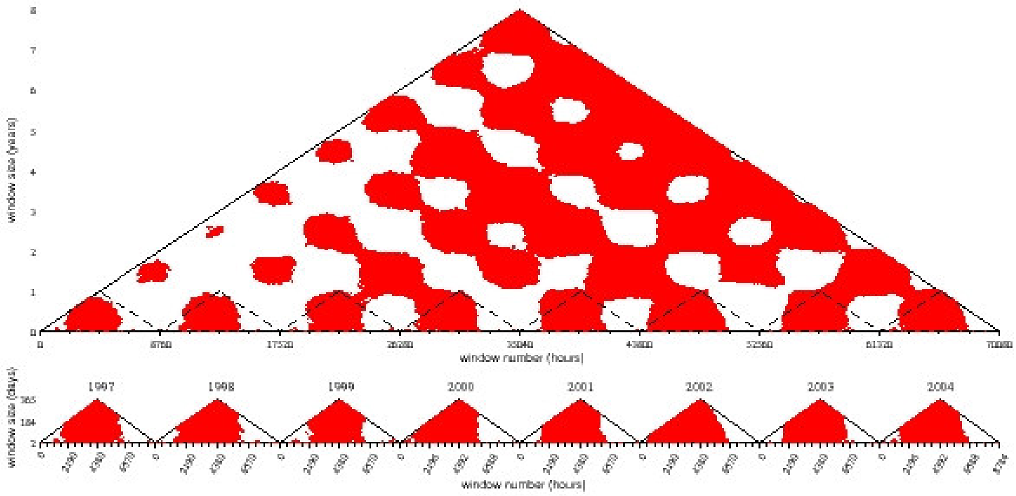

Figure A4 - Significant windows for whole periods periodic time series

SigWin-maps for a time series of hourly ozone ground level concentrations in Amsterdam, from 1997 to 2004 (5% mmFDR threshold level), made using SigWin-detector Config-Basic1. Upper: SigWin-map relative to the whole eight-year period. Lower: SigWin-maps relative to each year in the series.

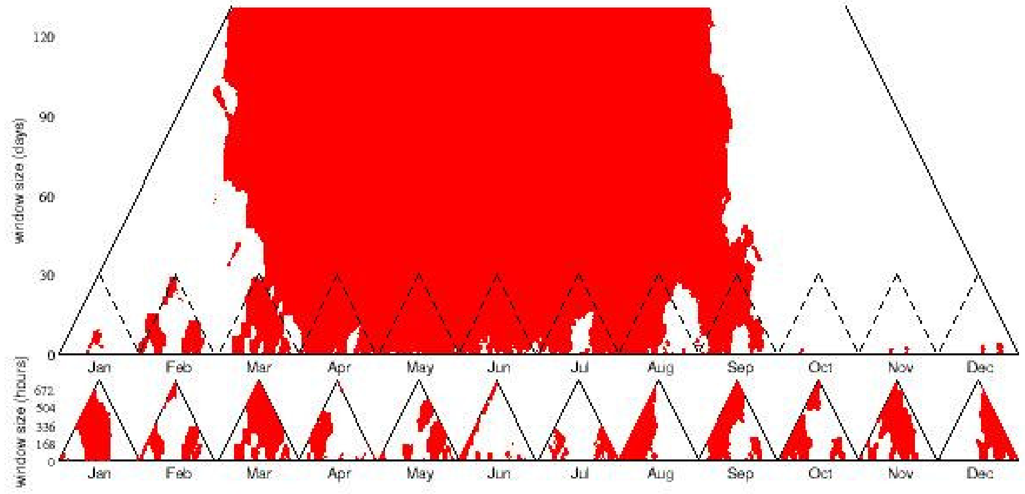

Figure A5 - Significant windows for incomplete periods periodic time series

SigWin-maps for a time series of hourly ozone ground level concentrations in Amsterdam, from 1997 to 2004 (5% mmFDR threshold level), made using SigWin-detector Config-Basic1. Upper: cropped version of the SigWin-map relative to 2004. Lower: SigWin-maps relative to each month in the year 2004.

References

1. **Airbase** [http://air-climate.eionet.europa.eu/databases/airbase/]
2. Inda MA, Belloum ASZ, Roos M, Vasunin D, de Laat C, Hertzberger LO, Breit TM: **Interactive Workflows in a Virtual Laboratory for e-Bioscience: the SigWin-Detector Tool for Gene Expression Analysis.** In *proceedings of the e-Science 2006; Amsterdam*. IEEE CS Press; 2006
