# Supplementary material for: SigWin-detector: a Grid-enabled workflow for discovering enriched windows of genomic features related to DNA sequences
Source: BMC Res Notes. 2008 Aug 8;1:63. doi: 10.1186/1756-0500-1-63 (PMC2533338; doi:10.1186/1756-0500-1-63)
Supplement: Additional file 5 — This tar file contains the source files of the WS-VLAM modules needed to run the SigWin-detector workflow, and some examples. To uncompress use. ▪ tar -xvzf SigWin-VLAM.v1.1.tar.gz (Linux users). ▪ WinZip or a similar tool. [file 1756-0500-1-63-S5.gz › SigWin-VLAM/SigWin-detector-Workflows.htm]

SigWin-detector alternatives


# Some SigWin-detector workflow configurations

            Here
we describe four alternative configurations for the SigWin-detector workflow.
Configurations Basic1 and Basic2 are used in the case we want to compute one single
SiqWin-map for the entire input data sequence. Configurations Sub1 and Sub2 are
used in the case that the input data sequence can be subdivided into logical
subsequences and we want to present the output in terms of those subsequences,
thus one SigWin-map per subsequence. For instance, a transcriptome map has
chromosomes as logical subsequences; a time series may have years, months,
days, etc, as logical subsequences. Configurations ending in 1 only compute one
significant window type: windows with significantly high median values or
windows with significantly low median value. Configurations ending in 2 compute
both significant window types and plot them with different colors.

## Contents:

SigWin-detector
Config-Basic: Detects significant windows in a sequence. 2

SigWin-detector
Config-Basic2: Detects significantly high median windows and significantly low
median in a sequence. 3

SigWin-detector
Config-Sub: Detects significant windows in a subsequence. 4

SigWin-detector
Config-Sub2: Detects significantly high median windows and significantly low
median windows for subsequences. 5

Module descriptions. 6

Input file example. 8

Output file example. 8

## SigWin-detector Config-Basic: Detects significant windows in a sequence.

### Input: A space-delimited file with (at least) one column containing the input sequence E= {*E*_1_, *E*_2_, …, *E_N_*}. A two-line header should precede the data. Input file example.

### Output:

1                   
A file containing the detected significant windows for
each label. Each data row represents a stretch of consecutive significant
windows. Column 1 gives the window size and columns 2 and 3 give the first and
last significant windows in the stretch. Output
file example.

2          An XMGRACE configurations file with
information on how to plot the resulting SigWin-map (Use: xmgrace -bat file.agrcmd)

### Graphical output: An XMGRACE plot displaying the resulting SigWin-map.

### Workflow:

### Parameters to set: (see module descriptions)

***ColumnReader****:* file\_name, column.

***SWMedian****:* min\_window\_size, max\_window\_size,
step\_size.

            ***FDRThresold:*** FDR\_level, threshold.

***SigWinSelect:***
Output\_file, threshold.

  

## SigWin-detector Config-Basic2: Detects significantly high median windows and significantly low median in a sequence.

### Input: A space-delimited file with (at least) one column containing the input sequence E= {*E*_1_, *E*_2_, …, *E_N_*}. Input file example.

### Output:

1       
      A file
containing the detected significantly high median windows for each label. Output file example.

2       
      A file
containing the detected significantly low median windows for each label. Output file example.

3       
      An XMGRACE
configurations file with information on how to plot the SigWin-maps. (Use:
xmgrace -bat file.agrcmd)

### Graphical output: An XMGRACE plot displaying the corresponding SigWin-maps.

### Workflow:

### Parameters to set: (see module descriptions)

***ColumnReader****:* file\_name, column.

***SWMedian****:* min\_window\_size, max\_window\_size,
step\_size.

            ***FDRThresold (upper):*** FDR\_level,
threshold=high. ***FDRThresold (lower):*** FDR\_level, threshold=low.

***SigWinSelect  (upper):*** Output\_file, threshold=high.
***SigWinSelect
(lower):*** Output\_file, threshold=low.

***StringJoiner:*** n\_streams=2

 

## SigWin-detector Config-Sub: Detects significant windows in a subsequence.

### Input: A space-delimited file with (at least) two columns. Column A: a sequence of labels L= {*L*_1_, *L*_2_, …, *L_N_*}. Column B: the input sequence E= {*E*_1_, *E*_2_, …, *E_N_*}. Input file example

### Output:

1                   
A file containing the detected significant windows for
each label. Output file example.

2          An XMGRACE configuration file with
information on how to plot the resulting SigWin-maps (Use: xmgrace -bat file.agrcmd)

### Graphical output: An XMGRACE plot displaying the resulting SigWin-maps.

### Workflow:

### Parameters to set: (see module descriptions)

***Read2Clumns****:* file\_name, column1, column2.

***SWMedian****:* min\_window\_size, max\_window\_size,
step\_size.

            ***FDRThresold:*** FDR\_level, threshold.

***SigWinSelect:***
Output\_file, threshold.

  

## SigWin-detector Config-Sub2: Detects significantly high median windows and significantly low median windows for subsequences.

### Input: A space-delimited file with (at least) two columns. Column A: a sequence of labels L= {*L*_1_, *L*_2_, …, *L_N_*}. Column B: the input sequence E= {*E*_1_, *E*_2_, …, *E_N_*}. Input file example.

### Output:

1       
      A file
containing the detected significantly high median windows for each label. Output file example.

2       
      A file
containing the detected significantly low median windows for each label. Output file example.

3       
      An XMGRACE
configurations file with information on how to plot the SigWin-maps. (Use:
xmgrace -bat file.agrcmd)

### Graphical output: An XMGRACE plot displaying the corresponding SigWin-maps.

### Workflow:

### Parameters to set: (see module descriptions)

***Read2Clumns****:* file\_name, column1, column2.

***SWMedian****:* min\_window\_size, max\_window\_size,
step\_size.

            ***FDRThresold (upper):*** FDR\_level,
threshold=high. ***FDRThresold (upper):*** FDR\_level, threshold=low.

***SigWinSelect
(lower):*** Output\_file, threshold=high. ***SigWinSelect (lower):***
Output\_file, threshold=low.

***StringJoiner:*** n\_streams=2

  

## Module descriptions

A short
description of each module containing the modules functionality, most used
parameters, input ports and output ports is given below. Only the parameters
that must be set before execution are listed (the other parameters may keep
their default value). The port connections are given in the workflow diagrams.
The contents of the input ports are the same as the contents of the
corresponding output ports. The ports are named by an abbreviation of the
module name followed by 'i' or 'o' (input or output respectively) and the port
number. Input ports are colored in blue and output ports in red. The ports are
numbered in the same order as they appear in the workflow diagrams. A detailed
description of the modules is given here.

| **Module functionality** | **Parameters** | **Input ports** | **Output ports description** |
| --- | --- | --- | --- |
| **ColumnReader**: Reads the input sequence **E****=** {*E*_1_, *E*_2_, …, *E_N_*}from a tab delimited file and transfers it to the output port. | *file\_name:* Input file name  *column:* Column number | CRi1 (Not used) | CRo1: A vector containing the input sequence **E** |
| **Read2Columns**: Reads two columns from a tab delimited file and transfers it to the output ports. Column A: a sequence of labels **L****=** {*L*_1_, *L*_2_, …, *L_N_*}. Column B: the input sequence **E****=** {*E*_1_, *E*_2_, …, *E_N_*}. | *file\_name:* Input file name  *column1:* Column A number  *column2:* Column B number | R2Ci1 (Not used) | R2Co2: A vector containing the labels **L.**  R2Co1: A vector containing the input sequence **E** |
| **SeqSplitter:** Computes labeled subintervals from **L.** A labeled subinterval is determined by consecutive labels with the same value. E.g., **L**={a, a, b, b, b} produces two subintervals *I*_1_= (a, 1, 2) and *I*_2_=(b, 3, 5), defined by the triple (label, start, end). | - | SSPi1 | SSPo1: A vector containing the labeled subintervals **I**={*I*_1_, *I*_2_, …}. |
| **Rank:** Computes the ranks **R****=** {*R*_1_, *R*_2_, …, *R_N_*} corresponding to **E.** | – | Ri1 | Ro1: The Rank structure corresponding to **E**.  Ro2: A vector containing **R**, a sorted version of **E**.  Ro3: A vector containing a sorted version of the non duplicate values of **E**. |
| **SWMedian:** Computes *m_S_*(*w*), the moving medians of **E,** for window sizes *S* = *S*_min_, *S*_min_+D*S*, …, *S*_max_ *=S*_min_+*q*D*S*. | *min\_window\_size: S*_min_  (odd)  *max\_window\_size:* Approximate value for *S*_max_  *step\_size:* D*S* (even) | SWMi1 | SWMo1: The parameters SW=(*N*, *S*_min_, *S*_max_, D*S*) corresponding to the sliding window structure.  SWMo2: A sliding window structure containing the computed moving medians (i.e., a sequence of vectors. Each containing *m_S_*(*w*), for *S* = *S*_min_, *S*_min_+D*S*, …, *S*_maz_). |
| **SWSplitter:** Splits the input sliding window structure. Each new substructure corresponds to a labeled subinterval. E.g., sliding window SW=(*N=30*, *S*_min_,=1, *S*_max_ *=15*, D*S=2*) and intervals *I*_1_= (a, 1, 12) and *I*_2_=(b, 13, 30) produce sliding window substructures SW_1_=(12, 1, 11, 2) and SW_1_=(18, 1, 15, 2). | - | SWSPi1   SWSPi2  SWSPi3 | SWSPo1: A vector containing the parameters for each sliding window substructure **SW**={SW_1_, SW_2_, …}.  SWSPo2: The corresponding sliding window substructures |
| **SWMedianProb:** Computes *f*_S_(*m*), the exact theoretical null hypothesis probability density function corresponding to the moving medians *m_S_*(*w*). |  | SWMPi1   SWMPi2 | SWMPo1: A sequence of vectors. Each containing *f*_S_(*m*),   for *S* = *S*_min_, *S*_min_+D*S*, …, *S*_min_+*q*D*S*. |
| **Sample2Freq:** Generates *g_S_*(*m*), the normalized frequency counts corresponding to the moving medians *m_S_*(*w*). |  | S2Fi2  S2Fi1 | S2Fo1: A sequence of vectors. Each containing *g*_S_(*m*),   for *S* = *S*_min_, *S*_min_+D*S*, …, *S*_min_+*q*D*S*. |
| **FDRThreshold:** Uses *g_S_*(*m*) and *f*_S_(*m*) to compute *m_k,S_* (or *m_j,S_*), the high (or low) mmFDR thresholds at a given level α. corresponding to each window size *S*, for *S* = *S*_min_, *S*_min_+D*S*, …, *S*_min_+*q*D*S*. | *FDR\_level*: α (0 to 1)  *Threshold:* high or low | FDRTi1   FDRTi2  FDRTi3 | FDRTo1: A sequence of high (or low) mmFDR thresholds *m_k,S_* (or *m_j,S_*),   one for each *S*. |
| **SigWinSelect:** Selects the windows for which the median value *m_S_*(*w*) is above (or below) the FDR threshold *m_k,S_* (or *m_j,S_*). The resulting significant windows are written to a tab-delimited file. | *Output\_file:* output file name.  *Threshold:* high or low | SWSi1   SWSi2   SWSi3 | SWSo1: Name of the file to which the resulting significant windows were written.  SWSo1: (Not used) |
| **StringJoiner:** Concatenate up to four strings and send then to the output port. In the SigWin-detector config-HT workflow: the strings are the names of the files containing the detected significantly high median windows and the significantly low median windows respectively. | *n\_streams:* number of input ports to use (1 to 4). | SJi1   SJi2  SJi3 (Not used)  SJi4(Not used) | SJo1: The concatenated string. |
| **SigWinPlotGrace** Generates an XMGRACE [13] configuration file with instructions of how to plot the resulting SigWin-map. |  | SWPGi1   SWPGi2 | SWPGo1: A file containing XMGRACE instructions on how to print   the resulting SigWin-map. |
| **XmGrace** Displays the resulting SigWin-map using XMGRACE. |  | XMGi1 | – |

 

## Input file example:

#size=26740
ncols=2 descr=hg18-htm

#chrom
expression

1       10.0

1       13.0

1       286.0

1       0

...

1       46.0

2       37.0

2       5.0

2       77.0

...

24      7.0

24      74.0

24      96.0

24      1.0

  

## Output file example:

#Windows
beneath FDRThreshold for hg18-htm:1

#printing
a point at wMin to get correct number of sets

1
1 1

#windowSize
first last

25
210 221

25
994 996

27
212 220

...

#Windows
beneath FDRThreshold for hg18-htm:2

#printing
a point at wMin to get correct number of sets

1
1 1

#windowSize
first last

29
18 21

47
30 31

49
29 32

51
29 32

...
